# Exploring the Impact of Gender and Income on Concerns Reported by Cancer Survivors Aged ≥85 Years in Canada: A Secondary Analysis of the Canadian Transitions Survey

**DOI:** 10.3390/curroncol33030142

**Published:** 2026-02-28

**Authors:** Lorelei Newton, Irene Nicoll, Fay J. Strohschein, Margaret I. Fitch

**Affiliations:** 1 School of Nursing, University of Victoria, Victoria, BC V8P 5C2, Canada; lorelei@uvic.ca; 2 Independent Researcher, Toronto, ON M4L 2K5, Canada; 3 Departments of Psychiatry, Medicine, and Community Health Sciences, Cumming School of Medicine, University of Calgary, Calgary, AB T2N 4N1, Canada; fay.strohschein@ucalgary.ca; 4 Bloomberg Faculty of Nursing, University of Toronto, Toronto, ON M4C 4V9, Canada

**Keywords:** older adults, aged ≥85 years, cancer survivorship, neoplasms, income, gender, patient-reported outcomes, national survey

## Abstract

This study describes concerns, positive experiences and suggestions for improvement in survivorship care from the perspectives of cancer survivors aged ≥85 years based on gender and income levels. A national Canadian survey was conducted in 2016 focusing on cancer survivors’ needs and experiences with follow-up care after treatment. This paper reports a secondary analysis drawn from the survey data reported qualitatively. In total, 581 respondents aged ≥85 years responded, of which 399 confirmed gender and annual household income. Within this group, 201 were male, and 198 were female. Two-thirds of the males (n = 134 males, 66.7%) and 80.8% of the females (n = 160) reported annual household income under $50K (CAD). Limited differences were noted between survivors’ responses according to sex and/or income levels. Concerns focused on physical challenges and body changes. Positive comments reflected appreciation of the care provided by attentive healthcare professionals. Suggestions for improvement addressed the need for improved person-centred care and availability of services for older adults. The survivors faced a range of physical, emotional and practical challenges following cancer treatment. The study highlights the importance of considering how living alone or with others, community connection, and the burden of co-morbidities compounding physical challenges after cancer intersect to create unique situations for this age group.

## Simple Summary

The number of older adults surviving cancer and its treatment is growing. However, cancer treatment leaves them with a range of challenges that can be difficult to manage. It is not clear if being male or female and the amount of financial resources one has makes a difference in the survivorship experience. Using written data from a nation-wide survey in Canada, the perspectives of 399 individuals in this age group were analyzed. They described concerns about physical issues and body image, positive experiences with attentive healthcare professionals, and recommendations for improvements in survivorship care about the need for services specifically organized for older adults. The study highlights the influence of living alone versus with others, community connections, and challenges with co-morbidities.

## 1. Introduction

Within the past several decades, the number of adults aged ≥85 years diagnosed with cancer has nearly tripled (an increase of 170%), and this group now comprises more than double the number of new cancer cases as compared to those aged 15 to 39 [1]. With advances in treatment and steady progress in overall cancer survival in Canada [2], the cohort of cancer survivors aged ≥85 years will continue to grow. It is expected that the number of adults in this age group diagnosed with cancer will more than triple again in the next 25 years (2022 to 2050, expected increase of 225%) [3].

Cancer treatment can leave survivors with physical, emotional, and practical consequences which influence their quality of life, despite advances in treatment and supportive care [4,5,6]. Many older adults live with co-morbid conditions, and coping with the aftermath of cancer treatment can add burden to living with an ongoing chronic illness and other age-related changes [7,8,9].

Treating individuals who have cancer in this heterogeneous age group, sometimes called the “oldest old”, is complex because of the higher likelihood of co-morbid conditions, declines in health status associated with aging, and the dearth of research regarding cancer treatment in this age group [10]. This population often deals with chronic conditions such as hypertension, osteoarthritis, heart disease, osteoporosis and chronic obstructive pulmonary disease (COPD) [11]. In addition, changes following cancer treatment are frequent, both physical [12,13,14] and emotional [15]. Depression, for example, among older patients with cancer rises with increasing age, being extremely common among the oldest old [16]. Lower levels of satisfaction with cancer care across domains of person-centred care are found among those aged ≥85 years [17]; however, less is known about the concerns and needs of this group during survivorship [17]. Greater understanding of the unique needs and concerns of this growing population with cancer is needed to inform the implementation of supportive care programs and services.

While Canada’s public healthcare system covers the financial costs of many valued healthcare services, other necessary services are only partially or not at all covered (i.e., medication, vision and dental care, allied health services, e.g., physiotherapy, occupational therapy, dieticians, psychological care, and transportation to access care). Canadian cancer survivors confront significant out-of-pocket costs [18]. Between 28% and 48% of adult survivors in Canada report financial burden due to direct (e.g., out-of-pocket) and indirect (e.g., lost income) costs as an unmet need [15]. Those living on a low income may have poorer health outcomes due to lack of nutritious foods, housing insecurity, and limited access to affordable transportation and non-insured health services [11]. This may be particularly relevant for seniors, many of whom are living on limited incomes. To date, little attention has been paid to the perspectives of cancer survivors aged ≥85 regarding income and sex.

Gender influences health and illness experiences throughout life and thus the way individuals cope with cancer [19,20]. The intersection of gender and income influences among older adults, however, has not been extensively studied in those aged ≥85 years. As this cohort is heterogeneous, there may be unique perspectives based on age, gender, and income. Examining differences in gender regarding challenges related to cancer survivorship in this age group may be helpful in highlighting and addressing possible needs, disparities and quality of life between males and females in this population and informing service development in cancer and community care.

## 2. Purpose

The purpose of this analysis was to describe the concerns, positive experiences after cancer treatment and suggestions for improvement in survivorship care reported by cancer survivors aged ≥85 years based on gender and income levels.

## 3. Methods

This work is a secondary analysis of qualitative data within the 2016 Transitions Survey Study in Canada. A full description and rationale for the parent study is in a previous publication [21]. In brief, the Experiences of Cancer Patients in Transition Survey was designed to capture the perspectives of cancer survivors one to three years following the completion of primary cancer treatment. It was designed by cancer experts and subjected to face and content validation by both healthcare providers and survivors. The survey was mailed to more than 40,000 individuals diagnosed and treated for cancer who were selected randomly from the ten provincial cancer registries. The eligibility criteria included adults (aged 30+ years) treated for breast, prostate, colorectal, and melanoma cancers with no metastatic spread, and selected hematological (e.g., Hodgkin’s lymphoma, diffuse B cell lymphoma, acute leukemia, acute lymphocytic leukemia) cancers; as well as adolescents and young adults (AYA, 18 to 29 years) with all non-metastatic cancer types except testes, where metastatic disease was included. Respondents could respond in English or French in hard copy mailed back to the investigators or through an online version. More than 13,000 individuals responded to the parent survey. These data are available from a publicly available national database [22].

Within the survey, three open-ended questions elicited feedback about the major concerns the respondent experienced, positive experiences or events which occurred during their cancer experience, and suggestions for improvements in survivorship or follow-up care. The specific questions were as follows: (1) What were the main challenges you experienced after you completed cancer treatment? (2) Reflecting on your experience with follow-up care after completing your treatment, please describe one positive experience you may have had that you think would benefit others going through a similar experience, and (3) Overall, what were the two most important things that could have been done that were not done to help you effectively deal with your needs after cancer treatment? Respondents wrote one or more responses to each question in narrative form.

Analysis: For the purposes of this secondary analysis, data for the respondents aged ≥85 years were extracted from the national database. Those who answered the annual household income question in the demographic section were included in the analysis. Respondents indicated if they were male, female, other (with option to specify) or “prefer not to answer”. The sample was divided into male and female and subsequently divided by income group (<$25,000; $25,000 to $49,999; $50,000 to $74,999; $75,000 and higher; CAD 2016 dollars). To allow for a distinct comparison, the lowest and highest income groups were isolated for analysis. Due to small numbers, those who indicated their gender as “other” or “prefer not to answer” were not included in the analysis. The final analysis was completed for four groups: female/low income, male/low income, female/high income, and male/high income.

Within each group, content analysis was conducted [23]. All investigators read through the written responses independently and devised content coding categories inductively. All investigators shared their perspectives and, through discussion, agreed upon content coding categories for each of the three questions. Two investigators (IN, MF) then coded all the written responses for each question using the agreed upon coding categories. The two investigators discussed their results to achieve consensus regarding the assignment of respondent comments to specific categories. The coded results were then discussed together with all research team members and a consensus reached about the observations to be reported. Chi square tests were used to identify statistically significant differences in the frequency of comments across the four income/gender groups and between income groups. *p* values < 0.05 were considered significant. Given the small sample size and the nature of the data, the orientation of the analysis and interpretation of findings became more about raising questions for future research study rather than informing definitive directions for clinical care.

Ethics approval was granted by respective ethics boards in the ten provincial cancer agencies participating in the original survey distribution. Participants signed consents prior to completing the survey. The original national survey data are housed in a publicly available platform with the Canadian Partnership Against Cancer.

## 4. Results

### 4.1. Demographic Characteristics

From the 12,292 national survey respondents, 581 were aged ≥85 years. Within this group, 1 respondent did not disclose gender; 5 respondents did not disclose either gender or income level; and 176 (106 female and 70 male) did not disclose income level. The 399 respondents who indicated gender as male or female and who answered the annual household income question were included in this secondary analysis.

Within the ≥85-years -of-age group, 198 were female, and 201 were male (Table 1). The majority of males reported being married or partnered 194 (71.6%) and 59 (21.8%) widowed compared to females, of whom 59 (19.4%) were married or partnered and 211 (69.4%) widowed. Close to 90% reported being retired. Of those who disclosed income (74.2% males and 65.1% females), 134 males (66.7%) and 160 females (80.8%) reported annual household income under $50K.

A comparison of the numbers of comments to blank responses and comments that did not address the survey questions is presented in Table 2. A summary of the codes generated for each question and their frequency are included in Table 3. The frequencies of codes by domain, sex and income, and comparison across income/gender groups appear in Table 4.

### 4.2. Main Challenges

Overall, 181 of the 399 respondents reported a total of 214 main challenges (see Table 2). One hundred and twenty-eight respondents in the under-$50K income level (70 females, 58 males) reported 149 main challenges; in the $50K-and-over income level, 53 respondents (34 males, 19 females) reported 65 main challenges. A slightly higher percentage of respondents (56.7% males, 56.9% females) in the under-$50K income compared to those in the $50K-and-over income level (50.7% males, 52.6% females) had blank or no responses to the question about main challenges.

Two-thirds of the main challenges reported were related to physical concerns (see Table 3). The most common physical problems were bladder and bowel issues (12.5%), reduced mobility (12.9%), and fatigue (11.4%). Descriptions included “poor bladder control and discomfort” (Male), “weakness in legs and balance (issues)” (Male), managing “daily routine activities as personal care, walking” (Male), “limited movement after mastectomy” (Female), and “limitation of my physical strength, hair loss, disinterest in social life—mentally overwhelming” (Female). Many of the comments concerned the difficulty of coping with stoma care and changing bladder and bowel habits, adjusting to limited mobility and stamina and regaining strength. Males and females in both income levels described similar physical issues. Significant differences in the number of main concerns identified were observed between the income groups (see Table 3).

In total, about a fifth (n = 42) of the main challenges were identified as emotional (see Table 3). Fear of recurrence (35.7%) and coming to terms with body changes such as hair loss and appearance of scars (26.2%) were mentioned most frequently. Relatively few main challenges were identified in the service delivery (10.8%), lifestyle adjustment (4.2%), or practical (3.7%) domains. Access to information regarding one’s condition and test results were mentioned most frequently as a main service delivery challenge. Males reported more physical challenges (55.8% to 70.0%) compared to females (47.8% to 60.8%) and more service delivery issues (11.6% to 14.3%) compared to females (4.3% to 11.4%). Females described more emotional challenges (22.8% to 34.8%) compared to males (11.4% to 18.6%). The only statistically significant difference was in the frequency of physical issues between the income groups.

### 4.3. Positive Experiences

Overall, 60.7% of the respondents did not answer the question; 194 respondents left the question blank and 48 indicated no positive experiences or made other comments that did not address the question (see Table 2). A total of 168 positive experiences were reported from the 157 respondents who provided an answer to the question (Table 3). In the under-$50K income category, 103 participants (54 females, 49 males) reported 110 positive experiences. In the $50K-and-over income category, 54 respondents (32 males, 22 females) reported 58 positive experiences.

Between 35.1% and 51.5% in each gender and income group reported positive experiences related to service delivery and healthcare professionals (Table 4). Males tended to describe satisfaction with care and treatment by the cancer centre or hospital generally, for example “good treatment”, “satisfied with care”, and “the care at the cancer clinic has been very good”. Females were more likely to comment about healthcare professionals specifically, including “a cancer specialist nurse planed and gave me her number to call if I needed assistance.”, “oncologist was expert, knowledgeable, professional, friendly, easy to communicate with” or “nurse during treatment was exceptional”. Many in all groups commented about the importance of support from family and friends; many also responded with comments/advice to others, i.e., “have hope, patience, trust”; “communicate with health care professionals” and “cooperate with the healthcare team”. Respondents in the under-$50K income level had a higher percentage of blank or no responses, (63.4% males and 65.0% females), compared to those in the higher income level (53.7% males and 47.4% females).

### 4.4. Suggestions for Improvement

Only 91 suggestions for improvement were submitted by 71 (17.8%) of all respondents (Table 2). Forty-six (11.5%; 30 females and 16 males) respondents in the under-$50K income group submitted 58 suggestions and 25 respondents (2.1%, 16 males, 9 females) in the $50K-and-over income group made 33 suggestions.

Over half of the 91 suggestions focused on improved service delivery and practical support (37.4%) or enhanced information and communications with patients and among healthcare professionals (27.5%) (Table 3). Respondents’ comments included “good help when problems occurred not available. A lot of sympathy but no real help.” (Male); “during/after cancer treatment tired easy, no social worker provided; cannot cook/clean after myself.” (Female); and needed “home care assistance that was reliable and consistent” (Female). Examples of comments addressing information and communication gaps were “more information and personal care from oncologist—I felt like I was just a number” (Female); “an opportunity to discuss the ’hand out’ pamphlet information” (Female); “need to talk to someone about a diagnosis—history of similar cases, feel very alone!” (Female); and “cancer doctor was off standish (*sic*); used medical terms until told him to speak in ’Layman’s’ terms” (Male). Male and female respondents reported similar issues. Slightly more respondents in the under $50K income level commented on practical issues such as transportation assistance and help with domestic chores. A higher percentage of those in the under $50K income level (88.1% males, 81.3% females) made no suggestions for improvement compared to those in the $50K-and-over income level (79.1% males, 78.9% females).

## 5. Discussion

This secondary analysis was conducted to explore the perspectives of cancer survivors aged ≥85 years about their major concerns, positive experiences and suggestions for improving survivorship care. This is the first study in Canada to explore these domains through the variables of gender and income levels for this older population of cancer survivors.

This age group of cancer survivors has not been studied extensively. The analysis of this broad Canadian sample offers some unique overall insights in an age group of community-dwelling cancer survivors—a population we expect to see more frequently in cancer care in the future. Although the sample is sufficient to explore the research questions of perspectives by gender and income, it is not large enough to perform other definitive sub-analyses. However, the data raises important questions for future exploration.

The proportion of cancer survivors aged ≥85 years who responded to the overall Transitions Survey (4.67%) is double the latest reported figures for the proportion in the Canadian population (2.3%) [24]. However, 4.67% is less than a quarter of Canadians aged ≥85 years who were diagnosed with cancer (estimate close to 18.0%) [25]. This suggests that adults aged ≥85 years may be under-represented in the Transitions Survey sample, possibly reflecting increased barriers to survey completion in this age group, such as increased sensory deficits, cognitive challenges, and/or fatigue. The proportion of males and females and the income levels also vary. Within the Canadian population, the ratio of women to men aged ≥85 years is gradually decreasing and currently is reported as 1.7 to 1. In our sample, the ratio was 1.1 women to men. The proportion of males to females in our sample, however, is fairly consistent with that among individuals aged ≥85 years diagnosed with cancer in 2016 (0.9 ratio of males to females).

The proportion of respondents with reported household incomes under $50K (73.6%) was higher compared to the Canadian population (51.1%) [26]. Although there is no definitive cut point for poverty level given the various factors that need to be considered (e.g., living location, people in household, community services and supports available), $25K or less for a single person is considered below the poverty line in most of Canada [27]. In our sample, 30.8% were in the 25K or less category, which closely approximated the Canadian population figure of 28.7%. Canada is considered to have one of the lowest levels of poverty; unfortunately, we are unable to contrast our sample to the population of survivors in a similar age group due to the nature of our data.

Other demographic characteristics which vary in our sample are the number of married men (71.6%) versus married women (21.8%) and the number of widowed men (19.4%) versus women (69.4%). Overall, 31.7% of Canadians aged ≥85 years are married, and 67% are not in co-habiting relationships [28]. The rates of widowhood are 31.9% for men in this age group and 69.2% for women. Given the economic implications for widowhood and the potential access to social support through a partnered relationship, these dynamics could be most important for cancer survivors in their management of recovery, especially those living independently in community settings and the emphasis across the country to have seniors remain at home as long as possible. Additionally, there is evidence to support an association between marital status and survival among people with cancer, with being married associated with better overall and cancer-specific survival [29,30,31], highlighting a need for more research regarding the impact of marital status or cohabitation/partnership for survival and quality of survivorship among older adults with cancer, and tailored supports and services to address this disparity.

Older adults constitute one of the fastest-growing age groups in the country and are expected to triple in the next 25 years [32], with a corresponding increase expected in the number of Canadians aged ≥85 years diagnosed with cancer [3]. With improvements in treatments, we can expect to see more of these oldest old survivors in our cancer centres. Presently, adults over 65 years account for more than 60% of the cancer diagnosed [1]. When a cancer diagnosis is made, treatment needs to be tailored appropriately to an older person’s health and functional status, based on geriatric assessment [33], to optimize quality and quantity of life in accordance with the person’s values and preferences [11,34]. Treatment decision-making may take on a different perspective considering how the oldest old might define quality of life and independent living versus younger cohorts [35], particularly if the older adult experiences mild cognitive impairments [36].

Unsurprisingly, the highest frequency of challenges cited by this sample of older adults were physical. Survivors are not only living with previous chronic illnesses, which may or may not have been exacerbated by cancer and its treatment, but could also be facing the normal physical declines of aging. These normal declines may be extenuated and occur faster than expected following a cancer diagnosis [37,38,39]. Adjustments in lifestyle because of mobility limitations, managing practicalities of wound care or stoma care, or performing other personal care activities can be particularly challenging for older adults following treatment that has predominate aftereffects of fatigue and functional or bodily changes [40].

Of note, the only significant difference between the income groups is related to physical issues. The number of physical issues mentioned was statistically higher for those with household income under $50K. This finding is consistent with other research where individuals with lower resources have concerns about being able to access assistance for their difficulties [41,42,43]. Astute assessment of a survivor’s capabilities and home support is essential prior to the end of treatment and discharge to home communities and may be promoted through integration of comprehensive geriatric management.

Responses regarding positive experiences and suggestions for improvement in care were very similar to those described in other survivors’ reports [44,45,46]. The importance of good communication, access to information and compassionate interactions with health care professionals have been cited repeatedly [47]. Access to mental health services, better coordination regarding follow-up care, and help in the home were seen as important by these older adults. These issues have also been cited by other cancer survivors and yet remain challenges in our cancer care system. Since the pandemic, availability of services has been reported as reduced in communities across Canada, and the healthcare sector continues to report shortages in personnel [48,49]. Although virtual programs have been developed, concern remains about how user-friendly these programs are for oldest old survivors [50].

One particularly intriguing observation for our team was the number of individuals who did not respond to the questions. Overall, those aged ≥85 years had a significantly higher percentage of blank or responses such as “none” or “no challenges” than all survey respondents in the Canadian Transition Survey [51,52,53]. Specifically, the percentage of blank/no responses regarding main challenges for this age group at all income levels was 59.6% compared to 35.7% for all survey respondents and was 59.6% compared to 12.8% for positive experiences for all survey respondents; close to 86.0% had no suggestions for improvement compared to 21.5% for all survey respondents.

We were somewhat surprised by so many non-responses. Given the age group and comorbidities, we expected to see more challenges and suggestions for improvement written. Providing written responses may have posed challenges due to physical limitations (e.g., eyesight, dexterity holding a pencil); sensory or cognitive difficulties or fatigue that interfered with writing clear descriptions; or feeling dismissed in the past and seeing little point in responding to questions. Potentially, some may feel they were not well enough informed about the health system to comment on it or simply associate their challenges with aging and not the aftermath of cancer. Low engagement with the survey may also reflect a broader disinterest in engaging with non-human interfaces for data collection. Future work is necessary to explore alternative ways of administering questionnaires (e.g., phone calls, one-to-one interactions) and ideas to help understand the validity of the survey responses from this age group.

### 5.1. Limitations

Our sample comes from across Canada, and thus, individuals are accessing health care from different provincial health care systems. Although the findings provide general insights, the concerns and suggestions for improvement may vary based on the services and resources available in various regions. Further research within specific jurisdictions is needed to inform the development of services and resources tailored to the context.

The written responses to the survey tended to be brief, very few were in full sentences, and it was difficult in some cases to interpret without having the benefit of follow-up interviews with probes or opportunities for deeper investigation. Future work would likely benefit from using interview-based qualitative approaches.

Overall, the low number of respondents in the age group could be seen as a limitation. The survivors in the age group who did not respond may well have distinct needs and concerns that are not represented in this survey-based analysis. In addition, akin to unintentionally excluding older adults from participation in research activities such as clinical trials, limitations to research designs may result in inadvertent disengagement of this age group.

Confidentiality issues limited information about survivors that could be shared from the registries in the original survey distribution. Hence, there was insufficient detail to allow weighting of surveys to make them representative of all Canadian survivors. In future studies, asking about perceived sufficiency of income or perceived financial impact of cancer care may provide additional insight about financial impact.

Finally, this analysis only included adults in this age group who identified as male or female. Use of this heteronormative framework may fail to capture the broader landscape of those who are non-heteronormative identified. Gender-diverse adults in this age group may have unique concerns, positive experiences, and suggestions for improvement that are not represented here. It is important to provide particular attention to diverse groups in future gender analyses and avoid perpetuating under-representation of non-binary and LGBTQ+ cancer survivors.

### 5.2. Implications for Clinical Practice/Research

This study has important implications for clinical practice and future research. Specific attention must be given in clinical practice and research to elicit the needs and concerns of those aged ≥85 years, as the low response rates for the survey and questions left blank suggest they may encounter barriers to expressing concerns and may be less inclined to express their needs. This may require more time during clinic visits, supports to overcome hearing or vision difficulties, or alternative approaches such as phone calls before or after appointments.

The differences in marital status between males and females highlight the need to assess availability of social support, particularly for those who are unmarried or widowed. These findings also point to the compounding challenges of being unmarried or widowed along with financial constraints/poverty that may limit an older person’s options for necessary support during and after cancer treatment [54].

It is critical that addressing the physical concerns draws on the multidisciplinary care team, including geriatricians and allied health providers (physiotherapy, occupational therapy), who have expertise in management of the physical concerns expressed by older adults, including incontinence management and treatment, mobility concerns, fatigue and impact on activities of daily living and safety at home [55]. Systematic integration of geriatric assessment into care supports assessment of physical concerns, practical concerns related to changes in functional status, and social support [56].

The second largest area of concern expressed by respondents was emotional concerns. The quotes, particularly from females, highlighted the importance of relational aspects of care, including information and communication gaps which emphasized the need for discussing diagnosis and proposed treatment compassionately. Among older adults with cancer, psychosocial interventions may be the least addressed [56], yet there is a clear need for psychosocial support tailored to this age group.

Nurses are well positioned to address many of the concerns and improvements identified in this secondary analysis by survivors aged ≥85 years. This could include conducting a geriatric assessment, providing understandable information and compassionate communication, offering relational and psychosocial support, and coordinating links to community and supportive care services. Consideration needs to be given to goals of care for this group, as such tools or frameworks do not currently reflect the experiences of the ‘oldest old’. The interventions would need to be made within the context of a multidisciplinary team with organizational support [55,57,58,59].

Future research regarding this older age group is imperative. The secondary analysis raised questions about topic areas that are important to inform future practice. Investigation is needed regarding influences of stigma and agism in care; healthcare providers’ identification of needs of older adults; best approaches for relevant conversations (e.g., goals of care, values/preferences, decision-making) given sensory challenges and/or patterns of social interaction (e.g., silent generation) [60,61]; engagement of family members; impact of receiving clear plans of care with relevant information and connection links providing easy access to resources; and how best to incorporate geriatric assessment and management in daily practice with this ≥85 age group given improved communication in cancer consultations [62], and improved outcomes can be achieved among older adults receiving cancer treatment [63].

## 6. Conclusions

Respondents aged ≥85 years identified specific concerns in physical and emotional domains, providing evidence of unmet supportive care needs for this age group. Important areas of improvement include tailored services and resources to address the unique concerns of older survivors, with particular attention to potentially different needs by gender, marital status, and level of income. Additional research, including qualitative approaches, would enhance understanding of the needs identified and help providers to tailor solutions for this growing population of cancer survivors.

## Figures and Tables

**Table 1:** Respondents aged ≥85 years age and income level.

Survey Respondents ≥ 85 Years				
Income *	Males	Females	Total	Percentage
Less than $25K	38	85	123	30.8%
$25K to <$50K	96	75	171	42.9%
$50 to <$75K	35	18	53	13.3%
$75K to <$125K	21	12	33	8.2%
≥$125K	11	8	19	4.8%
**Total**	**201**	**198**	**399**	**100.0%**

* Income reported in 2016 Canadian Dollars.

**Table 2:** Respondents aged ≥85 years—total comments compared to blank/no responses.

Topic	N = 399 ≥ 85 Years			
	Number of Challenges, Experiences, and Suggestions Submitted/Respondents	Respondents Left Question Blank	Respondents Wrote ‘None’, ‘Not Applicable’ or ‘No’	Respondents Did Not Address the Question (e.g., ‘No Treatment‘, ‘No Follow-Up Treatment’)
Main challenges	214/181	146	52	20
Positive experiences	168/157	194	33	15
Suggestions for improvement	91/71	232	65	31

**Table 3:** Respondents aged ≥85 years—total responses by domain and concern regarding main challenges, positive experiences and suggestions for improvement.

Domain	Concern	Number	*%* of Issue	Total	*%* of Total
**Main Challenges *n = 214***					
**Physical**	Bladder and bowl issues	27	*20.5%*		
	Limited mobility	17	*12.9%*		
	Fatigue	15	*11.4%*		
	Regaining strength, weakness	13	*9.8%*		
	Managing daily activities	10	*7.6%*		
	Other concurrent health issues (shingles, heart, etc.)	10	*7.6%*		
	Side effects, ongoing symptoms	9	*6.8%*		
	Pain	6	*4.5%*		
	Other (stomach, wound, etc.)	25	*18.9%*		
				**132**	*61.7%*
**Emotional**	Fear of recurrence	15	*35.7%*		
	Accepting body changes (scars, loss of hair)	11	*26.2%*		
	Accepting the disease	7	*16.7%*		
	Maintaining positivity, self-confidence, social engagement	5	*11.9%*		
	Other (e.g., mental health)	4	*9.5%*		
				**42**	*19.6%*
Service Delivery *	Information/communication about condition, test results	10	*43.5%*		
	Proper follow-up care, issues with appointments	8	*34.8%*		
	Home care issues	5	*21.7%*		
				**23**	*10.8%*
**Lifestyle Adjustments**	Adapting to new status, e.g., limited or changes to activities	7	*77.8%*		
	Finding suitable food, clothing	2	*22.2%*	**9**	*4.2%*
**Practical**	Return to work issues	2	*25.0%*		
	Caring for ill spouse	2	*25.0%*		
	Transportation	2	*25.0%*		
	Finding supplies, need for tests	2	*25.0%*	**8**	*3.7%*
**Total Main Challenges**				**214**	*100.0%*
***Positive Experiences n = 168***					
**Advice to Others**	Be positive, calm, enjoy life	19	*38.8%*		
	Communicate with, trust and follow medical team	13	*26.5%*		
	Have faith, trust	10	*20.4%*		
	Other: seek good professionals, peer and community supports	7	*14.3%*		
				**49**	*29.2%*
**Healthcare** Professionals **	Good surgeons, oncologists, nurses, family physicians, staff	41	*100.0%*	**41**	*24.4%*
**Family and Friends Support**	Good support from family and friends	34	*100.0%*	**34**	*20.2%*
**Service Delivery**	Good treatment, care, access and accommodation by cancer centre	30	*88.2%*		
	Care close to home, CCS support	4	*11.8%*	**34**	*20.2%*
**Other**	Supportive therapies (exercise, Thai Chi, peer support, faith)	10	*100.0%*	**10**	*6.0%*
**Total Positive Experiences**				**168**	*100.0%*
**Suggestions for Improvement *n = 91***					
**Service Delivery**	Access to cancer centre services (e.g., mental health)	10	*29.4%*		
	Better follow-up care	9	*26.5%*		
	Reliable in-home care, calls	7	*20.6%*		
	Length/timing of appointments, tests	4	*11.8%*		
	Other (care closer to home, service in first language)	4	*11.8%*		
				**34**	*37.4%*
**Information/** **Communications**	Information about process, tests, medications, diet, etc.	21	*84.0%*		
	Better communication with patients, among HCPs	4	*12.0%*		
				**25**	*27.5%*
**Healthcare** **Professionals**	Access to attentive, kind and empathetic HCPs	11	*100.0%*		
				**11**	*12.1%*
**Other**	Peer support, supportive therapies, thinking positive	11	*100.0%*		
				**11**	*12.0%*
**Practical**	Assistance with household chores, transportation, etc.	10	*100.0%*		
				**10**	*11.0%*
**Total Suggestions for Improvement**				**91**	*100.0%*

* Service Delivery—location; administration and access to booking tests, results and services; overall operation of treatment centres. ** Healthcare Professionals—behaviour; competence; interpersonal experience with nurses, family physicians, specialists and allied health professionals.

**Table 4:** Responses by domain, sex and income level.

Domain	Males				Females					
	Income < $50K		Income ≥ $50K		Income < $50K		Income ≥ $50K		Chi-Square	*p* Value
**Main Challenges *n = 214***										
Physical	49	*67.1%*	24	*61.5%*	48	*60.7%*	11	*47.8%*	3.39	0.07
Emotional	8	*11.0%*	8	*20.5%*	18	*22.8%*	8	*34.7%*	0.0	1.0
Service Delivery	10	*20.4%*	5	*12.8%*	8	*10.1%*	1	*4.3%*	1.48	0.22
Lifestyle Adjustments	5	*6.8%*	-	*0.0%*	4	*5.1%*	1	*4.3%*	0.02	0.85
Practical	2	*2.7%*	2	*5.1%*	1	*1.3%*	2	*8.7%*	0.19	0.66
**Total**	**73**	*100.0%*	**39**	*100.0%*	**79**	*100.0%*	**23**	*100.0%*	3.91	0.48
**Positive Experiences *n = 168***										
Advice to Others	19	*35.9%*	7	*21.2%*	17	*29.8%*	6	*24.0%*	*0.004*	*0.95*
Healthcare Professionals	7	*13.2%*	7	*21.2%*	21	*36.8%*	6	*24.0%*	*3.29*	*0.07*
Family and Friends Support	14	*26.4%*	4	*12.1%*	9	*15.8%*	7	*28.0%*	*1.79*	*0.18*
Service Delivery	13	*24.5%*	10	*30.3%*	8	*14.1%*	3	*12.0%*	*0.83*	*0.36*
Other	-	*0.0%*	5	*15.2%*	2	*3.5%*	3	*12.0%*	*0.75*	*0.39*
**Total**	**53**	*100.0%*	**33**	*100.0%*	**57**	*100.0%*	**25**	*100.0%*	*1.15*	*0.28*
**Suggestions for Improvement *n = 91***										
Service Delivery	11	*42.3%*	9	*50.0%*	9	*26.5%*	5	*38.5%*	*0.09*	*0.76*
Information and Communications	7	*26.9%*	3	*16.7%*	10	*29.4%*	5	*38.5%*	*0.22*	*0.64*
Healthcare Professionals	2	*7.7%*	1	*5.6%*	7	*20.6%*	1	*7.7%*	*0.04*	*0.85*
Practical	2	*7.7%*	4	*22.2%*	3	*8.8%*	2	*15.4%*	*0.78*	*0.38*
Other	4	*15.4%*	1	*5.6%*	5	*14.7%*	**-**	***-***	*0.02*	*0.89*
**Total**	**26**	*100.0%*	**18**	*100.0%*	**34**	*100.0%*	**13**	*100.0%*	*1.78*	*0.18*

## Data Availability

Data are within a publicly available dataset from the Canadian Partnership Against Cancer. The dataset is publicly available online: https://www.partnershipagainstcancer.ca/transition-study (accessed on 30 January 2025).
